# Supplementary material for: Resolving the full spectrum of human genome variation using Linked-Reads
Source: Genome Res. 2019 Apr;29(4):635–45. doi: 10.1101/gr.234443.118 (PMC6442396; doi:10.1101/gr.234443.118)
Supplement: Supplemental Material [file supp_gr.234443.118_Supplemental_longranger-2.2.zip › longranger-2.2.tar.bz2!/lr2.2-public/lib/stan/cmdstan/stan/doc/api/html/stack__alloc_8hpp.html]

Stan: src/stan/memory/stack\_alloc.hpp File Reference


|  |  |
| --- | --- |
|  | Stan  2.5.0  probability, sampling & optimization |


- Main Page
- Related Pages
- Namespaces
- Classes
- Files

- File List
- File Members

All Classes Namespaces Files Functions Variables Typedefs Enumerations Enumerator Friends Macros Pages

- src
- stan
- memory

Classes |
Namespaces |
Functions

stack\_alloc.hpp File Reference

`#include <cstdlib>`  
`#include <cstddef>`  
`#include <sstream>`  
`#include <stdexcept>`  
`#include <stdint.h>`  
`#include <vector>`  
`#include <stan/meta/likely.hpp>`

Go to the source code of this file.

|  |  |
| --- | --- |
| Classes | |
| class | stan::memory::stack\_alloc |
|  | An instance of this class provides a memory pool through which blocks of raw memory may be allocated and then collected simultaneously. More... |
|  | |

|  |  |
| --- | --- |
| Namespaces | |
|  | stan |
|  | Probability, optimization and sampling library. |
|  | |
|  | stan::memory |
|  | (Expert) Allocate, free, and manage underlying system memory. |
|  | |

|  |  |
| --- | --- |
| Functions | |
| template<typename T > | |
| bool | stan::memory::is\_aligned (T \*ptr, unsigned int bytes\_aligned) |
|  | Return `true` if the specified pointer is aligned on the number of bytes. More... |
|  | |


---

[ Stan Home Page ]

*© 2011–2014,
Stan Development Team.*
